# Psychosocial factors affected by burden in family caregivers of people with Alzheimer's disease

**DOI:** 10.1590/1980-5764-DN-2023-0115

**Published:** 2024-06-24

**Authors:** Edivaldo Lima de Araujo, Shirley Silva Lacerda

**Affiliations:** 1 Faculdade Israelita de Ciências da Saúde Albert Einstein, São Paulo SP, Brazil.; 2 Hospital Israelita Albert Einstein, São Paulo SP, Brazil.

**Keywords:** Alzheimer Disease, Caregiver Burden, Caregivers, Mood Disorders, Quality of Life, Doença de Alzheimer, Sobrecarga do Cuidador, Cuidadores, Transtornos do Humor, Qualidade de Vida

## Abstract

The world's population is experiencing an aging process, which is resulting in an increase in diseases such as Alzheimer's disease. Consequently, more and more people need care, which can lead to overload and harm to their family's quality of life.

**Objective:**

Identify the psychosocial factors affected by the burden on family caregivers of people with Alzheimer's disease.

**Methods:**

Forty-nine family caregivers of people with Alzheimer's disease, from a city in Minas Gerais, Brazil, participated in the study. They filled out a form of sociodemographic variables, and answered the Burden Interview Scale (BI-Zarit), Quality of Life in Alzheimer's Disease Caregiver version (CQoL-AD), the Depression, Anxiety and Stress Scale (DASS-21), the Mindfulness and Awareness Scale (MAAS) and the Clinical Dementia Rating Scale (CDR).

**Results:**

All participants were female with an average age of 54.26 (±8.99). Daughters comprised 77.55% of the sample, and 34.69% were sole caregivers. The Bi-Zarit scale positively and significantly correlated with DASS-21 Depression (r=0.440; p=0.002), DASS-21 Anxiety (r=0.415; p=0.003), DAAS-21 Stress (r=0.583; p<0.001). On the other hand, it showed a negative correlation with MAAS (r=-0.429; p=0.002) and CQoL-AD (r=-0.533; p<0.001).

**Conclusion:**

This study demonstrates that family caregivers of people with Alzheimer's disease may be overloaded, and that the heavier the burden, the lower level of attention, the worse quality of life and the greater the possibility for the caretaker to present symptoms of depression, anxiety, and stress.

## RESUMO

A população mundial vive um processo de envelhecimento que está resultando no aumento de doenças, como a doença de Alzheimer. Consequentemente, cada vez mais pessoas necessitam de cuidados, o que pode gerar sobrecarga e prejuízos à qualidade de vida de seus familiares.

**Objetivo:**

Identificar os fatores psicossociais afetados pela sobrecarga em cuidadores familiares de pessoas com doença de Alzheimer.

**Métodos:**

Participaram do estudo 49 cuidadores familiares de pessoas com doença de Alzheimer, de um município de Minas Gerais, Brasil. Os participantes preencheram um formulário de variáveis sociodemográficas e responderam à escala de Sobrecarga (BI-Zarit), à escala de Qualidade de Vida na Doença de Alzheimer versão Cuidador (CQdV-DA), à escala de Depressão, Ansiedade e Estresse (DASS-21), à escala de Atenção Plena e Consciência (MAAS) e à escala de Avaliação Clínica de Demência (CDR).

**Resultados:**

Todos os participantes eram do sexo feminino, com idade média de 54,26 (±8,99). As filhas representaram 77,55% da amostra e 34,69% eram cuidadoras exclusivas. A escala Bi-Zarit correlacionou-se positiva e significativamente com a DASS-21 Depressão (r=0,440; p=0,002), DASS-21 Ansiedade (r=0,415; p=0,003), DAAS-21 Estresse (r=0,583; p<0,001). Por outro lado, mostrou correlação negativa com MAAS (r=-0,429; p=0,002) e CQoL-AD (r=-0,533; p<0,001).

**Conclusão:**

Este estudo demonstra que os cuidadores familiares de pessoas com doença de Alzheimer podem estar sobrecarregados e que, quanto maior a sobrecarga, menor o nível de atenção, pior a qualidade de vida e maior a possibilidade de apresentar sintomas de depressão, ansiedade, estresse.

## INTRODUCTION

The world population is going through an aging process. In Brazil, as in other developing countries, this is happening at an accelerated pace. In 2018, 19.2 million Brazilians were elderly, which represented 9.2% of the general population. The Brazilian Institute of Geography and Statistics (2019) estimates that the number of people over 65 will reach 58.2 million in 2060^1^.

This process results in an increase in the number of people with Alzheimer's Disease (AD), which is the most prevalent cause of dementia in the world. It is a neurodegenerative disease that progresses with cognitive decline and functional loss that will inevitably lead the patient to depend on formal (professional) or informal (family) caregivers for activities of daily living (ADL)^2,3^.

Family caregivers play a fundamental role in providing physical and psychological support to sick family members. They take on a wide range of responsibilities from assisting with ADLs, monitoring medications, and ensuring safety, to giving emotional support. However, the intensity of this care can generate significant burden, negatively impacting physical and mental health, and consequently worsening the quality of life of these people. In a cohort study, Dauphinot et al. evaluated the association of AD severity with caregiver burden and found a greater burden in more advanced stages^4^.

The family caregiver is often a woman. In 50 to 70% of the cases they are wives or daughters, faced with challenging situations such as accepting the diagnosis, adapting to this new condition, managing possible family conflicts and reprogramming their present and future^5–7^.

Family caregivers of dependent individuals have reported that they are often faced with an intense and constant workload that can generate stress, exhaustion, and social isolation, leading to neglect of their own physical, mental, social, and financial well-being^7,8^.

Current literature shows that around 65% of family caregivers of people with dementia have physical and mental illnesses, in addition to excessive use of alcohol and medications for depression, anxiety and insomnia. Such situations may be associated with their resilience and ability to adapt to the new reality, which requires dedication, responsibility, patience and selflessness^7–9^. Hellis and Mukaetova-Ladinska (2022) carried out a systematic review on the psychological effect of taking care of people with AD on informal caregivers, and detected an increase in burden and worsening of quality of life, in addition to high levels of anxiety and depression^10^.

The objective of this study was to assess a possible correlation between burden and depression, anxiety, stress, level of attention and the quality of life of family caregivers of people with AD.

## METHODS

We carried out a cross-sectional evaluation with forty-nine family caregivers of people with AD, from a city in Minas Gerais, Brazil, in the first semester of 2022.

This study was approved by the Human Research Ethics Committee of Faculdade Israelita de Ciências da Saúde Albert Einstein (CEP-HIAE), under number 5.363.559 and CAAE 54214921.4.0000.0071. All participants signed a free informed consent form.

### Instruments

#### Sociodemographic form

This form considered sexual orientation, age, marital status, degree of kinship with the patient, time spent providing care, whether the patient was the only caregiver, education and family income.

#### Burden scale interview (BI-Zarit)

The Alpha Cronbach coefficient of this scale is 0.87, and aims to assess caregiver burden. Validated by Scazufca^11^, the Brazilian version contains 22 items that correspond to health, social and personal life, financial situation, well-being and personal relationships. The questions have four answers with scores from 0 (never) to 4 (always), and higher scores translate into high overload^11^.

#### Quality of life in Alzheimer's disease scale, caregiver version (CQoL-AD)

This scale assesses quality of life through the domains of physical health, energy, mood, housing, memory, family, marriage, friends, you as a whole, ability to perform tasks, leisure, money, and life as a whole. The version validated in Brazil, 0.87 Cronbach Alpha coefficient, contains 13 items with 4 scores each, with 1 point being poor and 4 points being excellent. Higher scores show better quality of life^12^.

#### Depression, anxiety and stress scale (DASS-21)

It consists of subscales that assess symptoms suggestive of depression, anxiety and stress. The total number of points for each domain is 21, and the value found must be multiplied by 2. The final classification for each domain is normal, mild, moderate, severe or extremely severe. The Brazilian validation has an Alpha Cronbach coefficient of 0.92 (depression), 0.86 (anxiety) and 0.90 (stress)^13^.

#### Mindfulness and awareness scale (MAAS)

The scale has 15 questions with a Likert response from 1 (almost always) to 6 (almost never) and the total score varies from 15 to 90 points. Lower scores indicate reduced mindfulness and little awareness in the present moment. The Alpha Cronbach coefficient of the Brazilian version is 0.83^14^.

#### Clinical dementia rating (CDR)

With this instrument it is possible to carry out a global cognitive and functional assessment. According to the score, the person is classified as: no dementia (0), questionable dementia (0.5), mild dementia (1), moderate dementia (2) and severe dementia (3). The Brazilian version has 98.1% accuracy, 91.2% sensitivity, 100% specificity and 97.8% negative predictive value^15^.

### Statistical analysis

For the analysis of continuous variables, the mean, standard deviation, minimum and maximum, were calculated, whereas the percentage was calculated for categorical variables. To evaluate the correlation between the variables studied, we used the Pearson coefficient. The statistical program adopted was JASP TEAM (2022, version 0.16.4).

## RESULTS

Only women participated in the study, and the average age was 54.26 (±8.99), 77.55% (n=38) were daughters, 67.34% (n=33) married, 34.69% (n=17) were sole caregivers, and 55.10% (n=27) were dedicated to caring between 16 and 24 hours a day. Most of them, 34.69% (n=17), had a family income between two and four minimum wages, and 77.6% (n=38) had more than eight years of schooling.

On the BI-Zarit scale, 92% (n=45) of the participants obtained scores indicative of overload. The results are presented in Table 1.

**Table 1:** Burden of family caregivers according to the Burden Scale Interview (BI-Zarit).

	n	%
Absent	4	8
Mild	16	32
Moderate	24	50
Severe	5	10

Notes: 0–20: Absent; 21–40: Mild; 41–60: Moderate; >60 Severe.

As for mood, 65.31% (n=32) had scores suggestive of depression, 53.06% (n=26) of anxiety and 55.10% (n=27) of stress, as assessed by the DASS-21 scale. These results are detailed in Table 2. In the QoLAD-C, the domain with the highest score (good or excellent) was related to family, with 83.67% (n=41), and the worst score (poor or fair) was leisure, with 69.39% (n=34).

**Table 2:** Depression, Anxiety and Stress Scale (DASS-21) scores.

	Depression (%)	Anxiety (%)	Stress (%)
Normal	17 (35)	23 (47)	22 (45)
Mild	9 (18)	2 (4)	3 (6)
Moderate	11 (23)	8 (16)	12 (25)
Severe	4 (8)	5 (10)	8 (16)
Extremely severe	8 (16)	11 (23)	4 (8)

Notes: Depression: Normal 0–9, mild 10–13, moderate 14–20, severe 21–27, extremely severe >27; Anxiety: Normal 0–7, mild 8–9, moderate 10–14, severe 15–19, extremely severe >19; Stress: Normal 0–14, mild 15–18, moderate 19–25, severe 26–33, extremely severe >33.

The Bi-Zarit scale positively correlated with DASS-21 Depression (r=0.440; p=0.002), DASS-21 Anxiety (r=0.415; p=0.003), DAAS-21 Stress (r=0.583; p<0.001). However, it showed a negative correlation with MAAS (r=-0.429; p=0.002) and CQoL-Ad (r=-0.533; p<0.001). These results are detailed in Table 3.

**Table 3:** Pearson's correlations between burden and depression, anxiety, stress, attention and quality of life.

			CDR	BI-ZARIT	DASS 21			MASS
					Depression	Anxiety	Stress	
BI-ZARIT	r		0.149					
	p-value		0.306					
DASS 21	Depression	r	-0.077	0.440				
		p-value	0.598	0.002*				
	Anxiety	r	-1.108	0.415	0.692			
		p-value	0.462	0.003*	<0.001*			
	Stress	r	0.030	0.583	0.792	0.780		
		p-value	0.839	<0.001*	<0.001*	<0.001*		
MASS	r		-0.079	-0.429	-0.631	-0.496	-0.519	
	p-value		0.589	0.002*	<0.001*	<0.001*	<0.001*	
CQoL-AD	r		-0.011	-0.533	-0.629	-0.467	-0.538	0.534
	p-value		0.939	<0.001*	<0.001*	<0.001*	<0.001*	<0.001*

* Note: p<0.01. BI-ZARIT: Burden Scale Interview; DASS 21: Depression, Anxiety and Stress Scale; MASS: Mindfulness and Awareness Scale; CQoL-AD: Quality of Life in Alzheimer's Disease Scale, caregiver version; CDR: Clinical Dementia Rating Scale.

## DISCUSSION

As the population ages and AD cases progressively increase, more attention has been paid to the health of family caregivers. This study aimed to evaluate the possibility that the burden on caregivers of family members with AD correlates with their quality of life and level of attention, as well as symptoms of depression, anxiety and stress.

In line with the findings in the current literature^2,5–7,16,17^, our study showed that most caregivers were daughters, married, sole caregivers and had a family income between two and four minimum wages. We assume that, in addition to caring for the sick family member, these women have other responsibilities such as household chores and dedication to other family members, which can increase their physical, mental and financial burden and contribute to a worse quality of life.

As dementia evolves, the sick family member will experience worsened functioning and increased dependence on other people. Even though most studies show that the burden is heavier in more advanced stages of AD^17–19^, in this study there was no such difference. This result might be accounted for by the fact that most of our sample had moderate dementia and few of the participants had mild and advanced dementia.

In accordance with the cross-sectional studies by Manzini and Vale^20^ and Liu et al.^7^, we found that caregivers’ burden was positively and significantly correlated with symptoms of depression, stress and anxiety. Such results suggest that family caregivers are more prone to mental disorders, which can be explained by the high burden of care, social isolation, and neglect of their own health.

On the other hand, overload was negatively correlated with the level of attention and quality of life, similar to the findings of Canadians Mank et al.^21^, which demonstrated that caregiver partners, who spent more time on care, had greater burden and worse quality of life since the onset of the disease. This may result from the high demand for care, which limits free time for self-care, social interaction and leisure. The impairment of attention level may be related to the excess of chores, worsened sleep quality and mental disorders, when present.

The limitations of the study were the sample size, with only a few people with mild and advanced dementia and the majority in the moderate phase. Additionally, the design of this study does not make it possible to establish a causal relationship between the analyzed outcomes.

In conclusion, this study demonstrates that family caregivers of people with Alzheimer's disease may be overloaded, and that the heavier the burden, the lower the level of attention, the worse the quality of life and the greater the possibility of presenting symptoms of depression, anxiety, and stress. Therefore, more attention should be paid to this population. In this sense, interventions such as psychoeducation programs, access to the health system and professional guidance can be crucial strategies to support these family members.
